# Allogenic vs. synthetic granules for bone tissue engineering: an in vitro study

**DOI:** 10.1007/s40204-018-0092-3

**Published:** 2018-07-17

**Authors:** Farnaz Kouhestani, Farnaz Dehabadi, Mehrnoosh Hasan Shahriari, Saeed Reza Motamedian

**Affiliations:** 1 Department of Periodontics, School of Dentistry, Shahid Beheshti University of Medical Sciences, Tehran, Iran; 2 Department of Oral and Maxillofacial Surgery, Faculty of Dentistry, Mashhad University of Medical Sciences, Mashhad, Iran; 3 Dental Research Center, Research Institute of Dental Sciences, School of Dentistry, Shahid Beheshti University of Medical Sciences, Tehran, Iran; 4 Department of Orthodontics, School of Dentistry, Shahid Beheshti University of Medical Sciences, Tehran, Iran

**Keywords:** Bone regeneration, Tissue engineering, Bone substitute, Dental pulp stem cells, Tricalcium phosphate, Hydroxyapatite, Allograft, In vitro study

## Abstract

The aim of this study was to compare human dental pulp stem cells’ (DPSCs) attachment, proliferation and osteogenic differentiation on allogenic and synthetic biphasic bone granules. In this in vitro study, two types of bone granules were used: allograft [freeze-dried bone allograft (FDBA)] and biphasic granules [hydroxyapatite/beta-tricalcium phosphate (HA/β-TCP)]. By isolation of DPSCs, their attachment to bone granules was observed by scanning electron microscope (SEM) at day 1 and 7 of cultivation. Vital cells were measured by MTT assay at 1, 3, and 7 days of cell culture. Comparison of vital cells at different time points was considered as cell proliferation. Finally, differentiation of DPSCs was evaluated by measurement of alkaline phosphatase (ALP) activity 3, 7, 14, and 21 days after cell seeding in standard and osteogenic media. Data were analyzed using two-way ANOVA with a significant level of 0.05. Attachment of DPSCs on FDBA granules seemed relatively stronger. The number of cells (based on MTT values) and ALP activity of the cells cultured on both study groups increased between time points (*p* ≤ 0.001). FDBA granules had more cells compared to HA/β-TCP granules (*p* < 0.001). There was no significant difference between ALP activity of two study groups cultured in the standard medium (*p* = 0.347) and they were both higher than the control group (*p* < 0.05). In the osteogenic medium, FDBA group had significantly higher ALP activity compared to HA/β-TCP (*p* = 0.035) and control (*p* = 0.001) groups while there was no significant difference between ALP activity of HA/β-TCP and control groups (*p* = 0.645). In conclusion, current in vitro study revealed that FDBA granules have more potential in supporting DPSCs attachment and proliferation and inducing their ALP activity compared to HA/β-TCP granules. Therefore, FDBA could serve as a proper bone substitute material.

## Introduction

In orthopedics and oral and maxillofacial surgery, bone defects resulting from trauma, neoplastic and pathologic diseases, periodontitis, and congenital conditions are common treatment challenges. The “gold standard” treatment modality of these defects is autogenous bone grafts. However, harvesting these grafts is associated with several disadvantages such as donor site morbidity, limited size and shape, extra costs, and necessity of hospitalization (Khojasteh and Motamedian 2016; Jafari et al. 2017). Therefore, researchers try to find an alternative bone regeneration method. One of the proposed methods involves the use of bone substitutes in conjugation with stem cells to provide an engineered graft material with similar features to those of autogenous bone grafts (Khojasteh et al. 2015; Motamedian et al. 2015).

Scaffolds and bone substitute materials are used not only to mechanically carry the cells to the defect site, but also to provide a medium for stem cells to proliferate and differentiate to osteogenic cells (Malik et al. 1992). The literature reviews show that synthetic and allogenic scaffolds are among the most frequently used bone substitute materials in tissue engineering (Tabatabaei et al. 2012; Motamedian et al. 2016; Hosseinpour et al. 2017).

One of the commonly used synthetic bone granule is biphasic hydroxyapatite/tricalcium phosphate (HA/TCP) scaffold, the application of which has been investigated in in vitro (Arinzeh et al. 2005; Zhang et al. 2008; Cordonnier et al. 2010, 2014; Pripatnanont et al. 2016) and in vivo (Farina et al. 2008; Zhang et al. 2008; Cordonnier et al. 2014; Yang et al. 2014) studies. HA/TCP is a biocompatible material which poses osteoconductive features providing an environment for new bone formation in vivo (Arinzeh et al. 2005).

Allograft bone materials have been used in orthopedics since 1950s and their current preparation and sterilization methods reduce the risk of disease transmission and host immune response compared to fresh bone allografts (Carr and Hyatt 1955). It has been stated that allografts possess osteoinductive features for promoting new bone formation (Truedsson et al. 2013), as well as osteoconductive properties which help production of more new bone formation and cellular attachment and proliferation (Wei et al. 2015).

Despite the vast clinical use of bone substitute materials, there are limited in vitro studies evaluating the response of cells toward them (Tang et al. 2002; Arinzeh et al. 2005; Cordonnier et al. 2010; Seebach et al. 2010; Cordonnier et al. 2014, Lafzi et al. 2016). The aim of the current study was to compare the dental pulp stem cells’ (DPSCs) attachment, proliferation, and differentiation on two types of commonly used bone granules.

## Materials and methods

### DPSCs cultivation

Dental pulp stem cells were collected from the pulp tissue of impacted mandibular wisdom teeth of three young (20, 21 and 23 years old) healthy male adults. An informed consent was obtained before extraction of the teeth. The teeth were rinsed with phosphate buffered saline (PBS) (Sigma-Aldrich, St. Louis, MO, USA) and the pulp tissue was removed, following separation of the crown by dental forceps. The pulp tissue was digested by incubation in 3 mg/ml collagenase solution (Sigma-Aldrich) dispense in PBS at 37 °C for 1 h. Cell suspension was then centrifuged and the cells were immersed in standard culture media [DMEM (Life Technologies, Carlsbad, CA, USA), 20% FBS (Invitrogen, Carlsbad, CA, USA) and 1% penicillin/streptomycin 10,000 µ/mL (Life Technologies)] and placed in 25 cm^2^ flasks and incubated at 37 °C and 5% CO_2_. DPSCs were passaged three times using 0.25% trypsin plus EDTA (Life Technologies) before being used in this study.

### Flow cytometry

High expression of mesenchymal stem cell markers (CD44, CD90, CD73, and CD105) (Abcam) and low expression of hematopoietic cell markers (CD34 and CD45) (Abcam) by cultivated cells were assessed by flow cytometry. Briefly, fluorescein isothiocyanate (FITC)-conjugated monoclonal antibodies against these markers were assessed by flow cytometer (Partec, Germany). A concentration of 2 μg/mL of antibodies was applied at 4 °C for 30 min. Then, cells were rinsed with PBS and fixed with paraformaldehyde 4%.

### Differentiation potential

To evaluate the differentiation potential of the harvested cells, third passages were cultured in osteogenic (DMEM plus 0.2 Mol/L ascorbic2-phosphates, 10^−8^ Mol/L dexamethasone, and 10 mMol/L β glycerol phosphate) and adipogenic (DMEM plus 0.2 mMol ascorbic2-phosphate, 10 mMol beta-glycerophosphate, and 50 mg/mL indomethacin) media. To assess the osteogenic and adipogenic differentiation potential, cells were fixed with paraformaldehyde 4% for 10 min and stained by alizarin red and oil red solutions for 15 min, respectively.

### Cell seeding

In this in vitro study, macroporous biphasic calcium phosphate (MBCP) 1–2 mm granules (Biomatlante SA, Vigneux de Bretagne, France), which is a biphasic HA/β-TCP material and particulate cortical/cancellous mineralized FDBA 1–2 mm granules (Tissue Regeneration Co. Tehran, Iran), were evaluated. Six samples were used for each group at each time point and each test. 20 mg of granules was placed in 48-well plate and a cell suspension including 2.5 × 10^4^ DPSCs (200 µL cell suspension with a concentration of 1 × 10^5^ cells) was seeded to the wells. Plates were incubated for 1 h at 37 °C and then standard or osteogenic media were added to the wells based on the test. The osteogenic media were used only in half of the samples which were prepared to alkaline phosphatase (ALP) activity assay. Finally, the plates were incubated at 37 °C and 5% CO_2_.

### Scanning electron microscope

To view the attachment of DPSCs on the surface of the bone granules, scanning electron microscopy (SEM) was used. After 1 and 7 days of cultivation, cells were rinsed with PBS and fixed for 2 h in 2.5% glutaraldehyde and 1 h in 1% osmium. Then, the samples were dehydrated by ethanol and dried in a desiccator overnight. Following sputtering with gold, the samples were observed with SEM (VEGA, TESCAN, Czech Republic). Also, the sizes of porosities in the images were measured by ImageJ computer software v.1.46 (Wayne Rasband (NIH)).

### MTT assay

To assess cell viability, MTT assay was performed in all groups 1, 3, and 7 days after cell seeding. Briefly, culture media were removed and cells were incubated with 10% MTT ((3-(4,5-dimethylthiazol-2-yl)-2,5-diphenyl (tetrazolium bromide, Sigma-Aldrich)) for 4 h at 37 °C. Then, they were incubated with dimethyl sulfoxide (Carlo Erba, Italy) overnight. The supernatants were moved to 96-well plate and absorbance was read using microplate reader (Anthos, Austria) at 590 nm wavelength.

### ALP activity

Alkaline phosphatase activity was measured 3, 7, 14 and 21 days after cell seeding. For this test, half of the samples was cultured in the standard media and the other half was cultured in the osteogenic media. Briefly, samples were rinsed twice with PBS and homogenized in lysing buffer (pH 7.5, 10 mM Tris–HCl, 1 mM MgCl_2_, and 0.05% Triton X-100). Then, *p*-nitrophenol phosphate (PNP) (Sigma-Aldrich) and alkaline buffer (Sigma-Aldrich) was added to the lysed cells and incubated for 15 min at 37 °C. The reaction was stopped using 0.5 N NaOH. The absorbance was read using microplate reader (Anthos) at 405 nm wavelength.

### Statistical analysis

Comparison between study groups and culture time points was performed using two-way ANOVA followed by Tukey post hoc test. Data were analyzed using SPSS v.18 computer software (SPSS, Chicago, IL, USA) at a significance level of 0.05.

## Result

### Stem cells

Stem cell markers including CD44, CD90, CD73, and CD105 were expressed in 99.7, 100, 96.1 and 99.9% of the cells while hematopoietic markers including CD45 and CD34 were expressed in 0.438 and 0.662% of the cells. Also, alizarin red and oil red staining showed osteogenic and adipogenic differentiation potential of the cultured cells.

### SEM

**Fig. 1:**
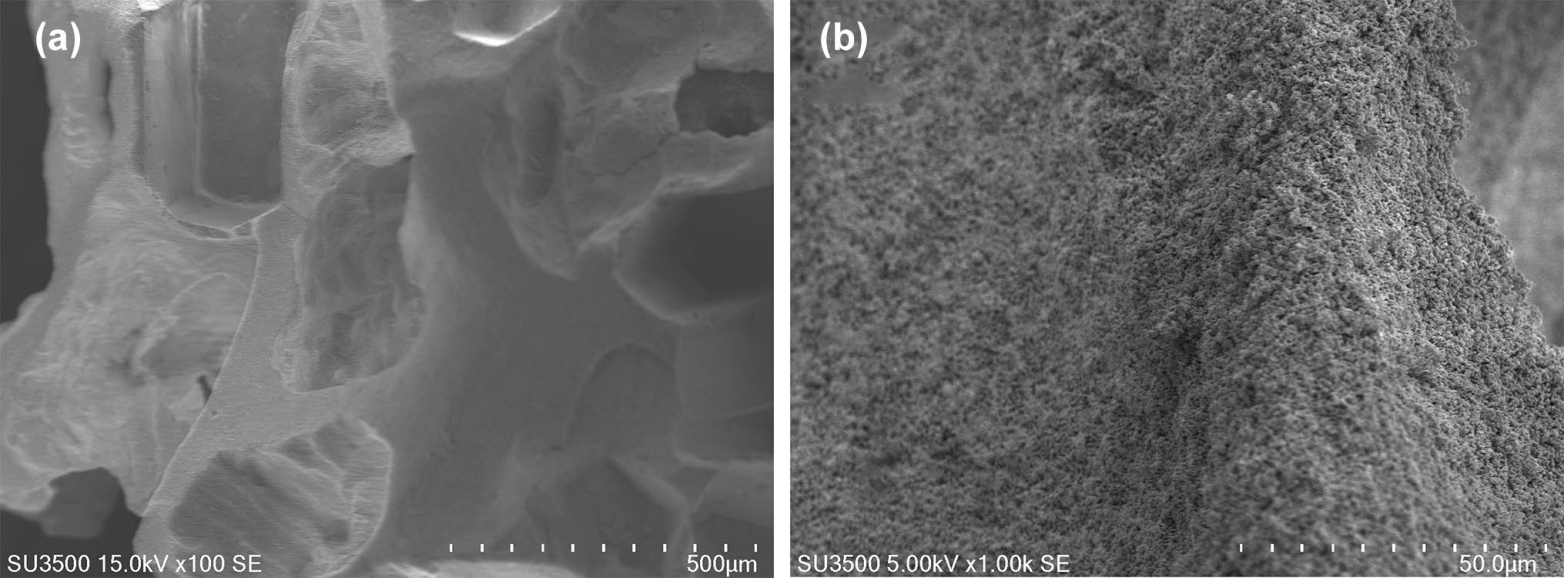
SEM image of the surface of unloaded MBCP granules **a** × 100 and **b** × 1000

**Fig. 2:**
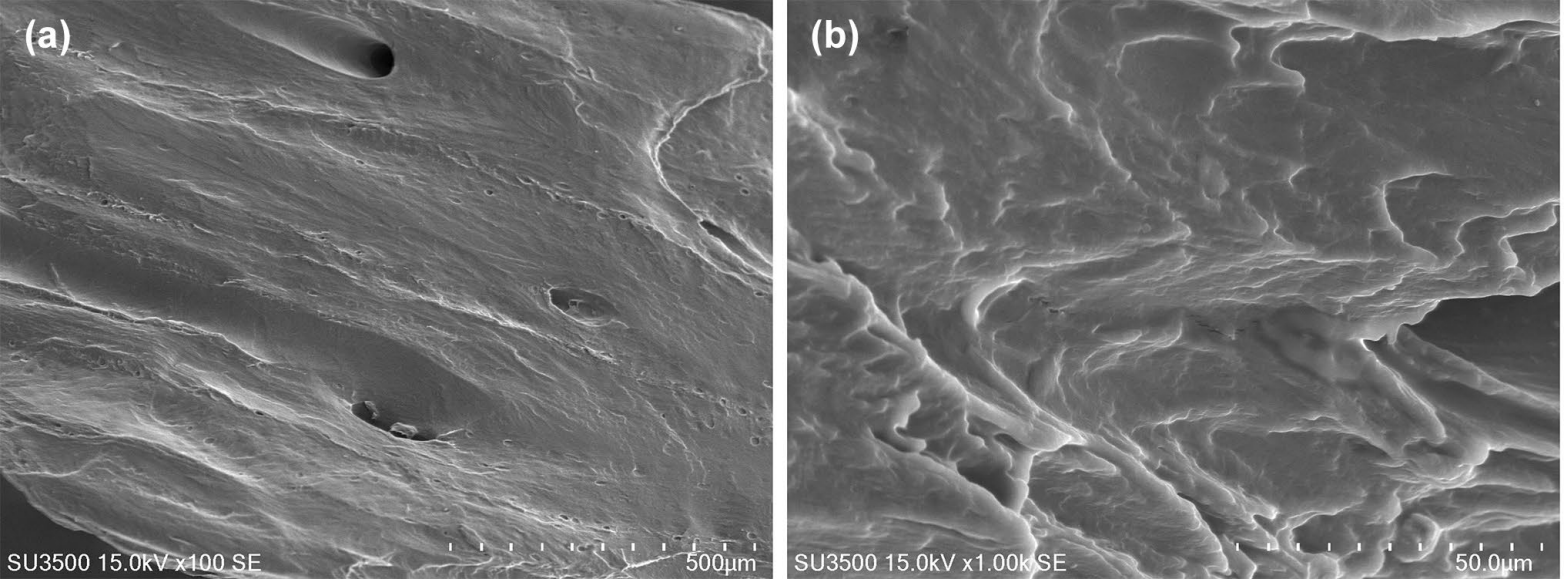
SEM image of the surface of unloaded FDBA granules **a** × 100 and **b** × 1000

The MBCP granules were made of small particles with 5–15 µm diameter and 20–50 µm height. Unloaded granules had 350–700 µm macropores which did not seem to be interconnected as well as 2–20 µm micropores (Fig. 1). The FDBA granules had relatively few 50–500 µm pores and they had a lamellar texture (Fig. 2).

**Fig. 3:**
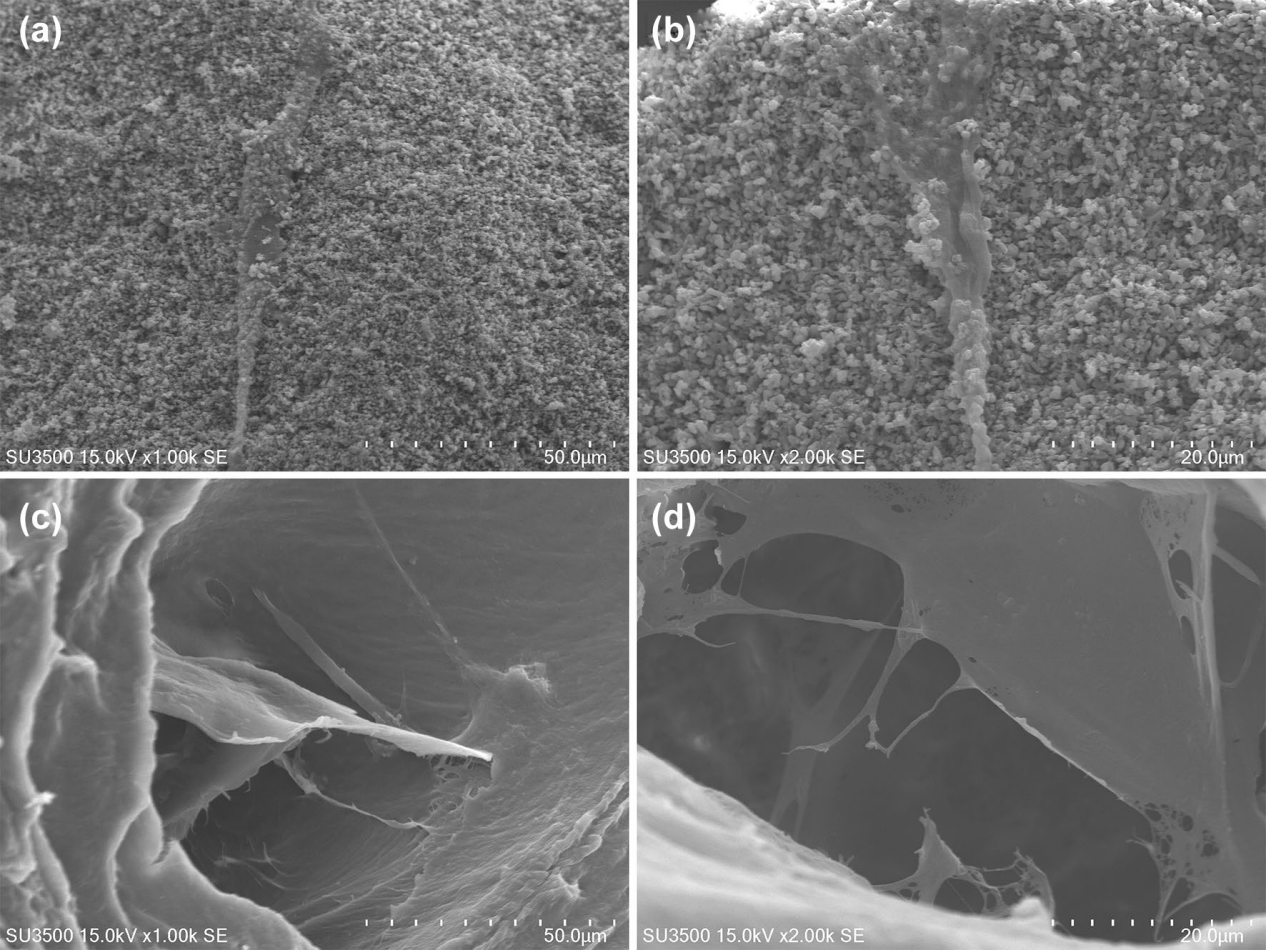
Attachment of hDPSCs on the surface of **a**, **b** MBCP granules and **c**, **d** FDBA granules; 1 day after cell seeding

**Fig. 4:**
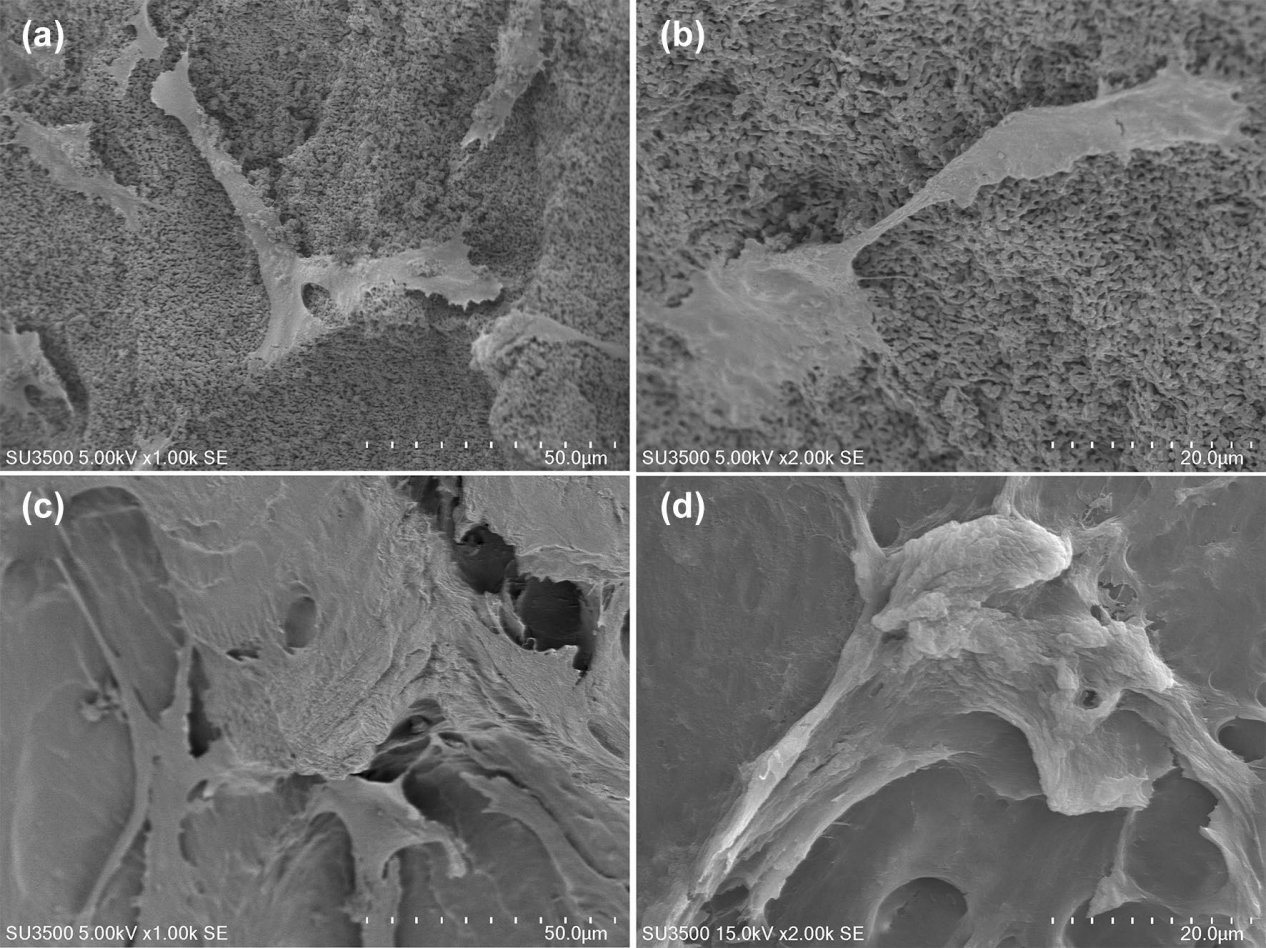
Attachment of hDPSCs on the surface of **a**, **b** MBCP granules and **c**, **d** FDBA granules; 7 days after cell seeding

One day after cell seeding, DPSCs loaded on FDBA seemed to have stronger adhesion and produced extracellular matrix (ECM) compared to DPSCs loaded on MBCP (Fig. 3). Seven days after cell seeding, the population of attached cells was increased in both groups while stronger attachment of cells on FDBA by their pseudopods was observed (Fig. 4). Attachment of stem cells on bone granules was not quantified in the SEM images.

### MTT assay

**Fig. 5:**
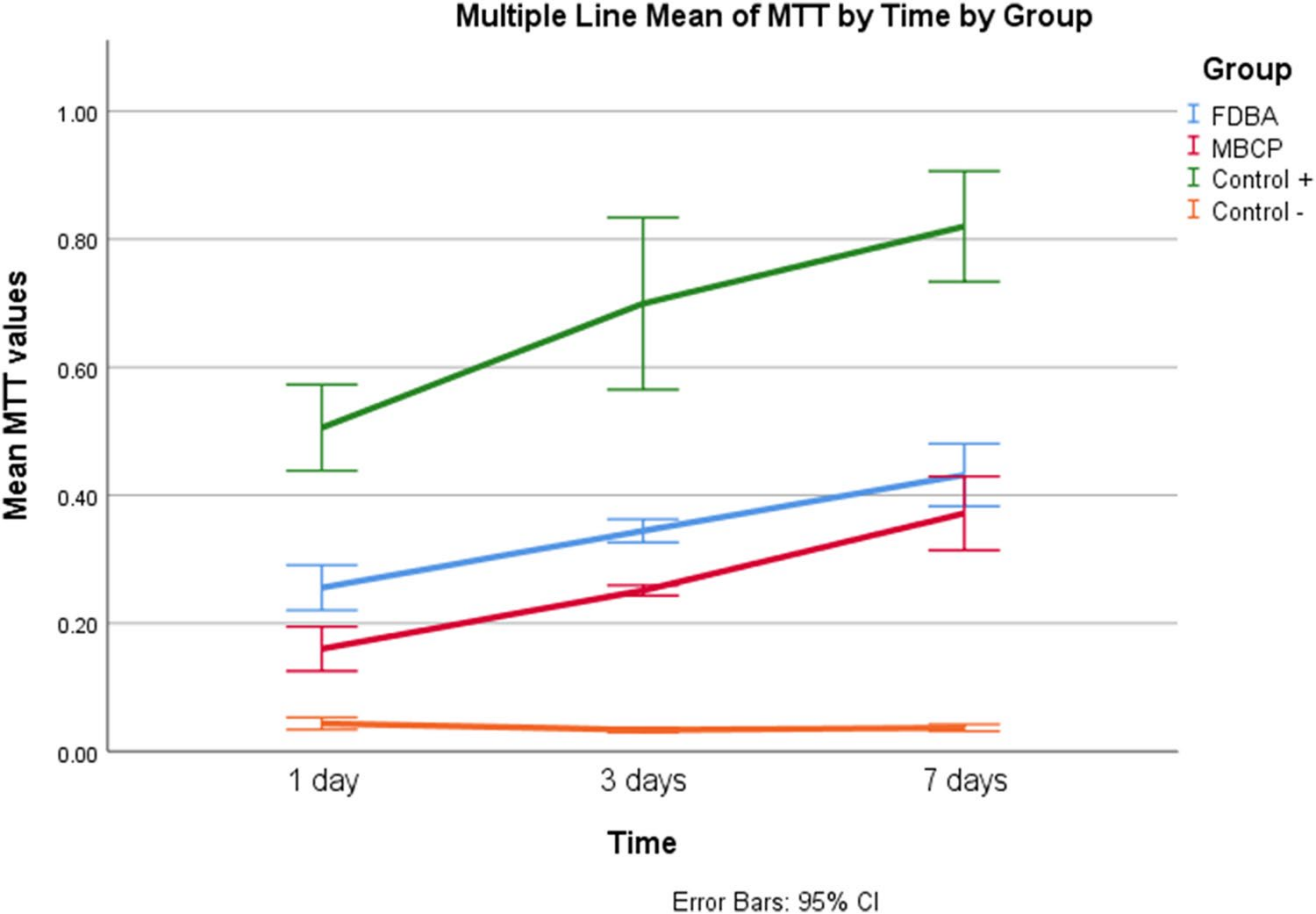
Comparison of mean MTT values between study groups at different time points

Statistical analysis showed that the group and the time, as well as their interaction, had a significant effect on MTT values (*p* < 0.001). As demonstrated in Fig. 5, the mean MTT values in all the groups except for the control group were increased significantly from day 1 to day 7 of culture (*p* < 0.001). The highest mean MTT value belonged to control + group (*p* < 0.001). Comparing two types of bone granules showed that MTT values of FDBA group were significantly higher than those of MBCP group (*p* < 0.001).

### ALP activity

**Fig. 6:**
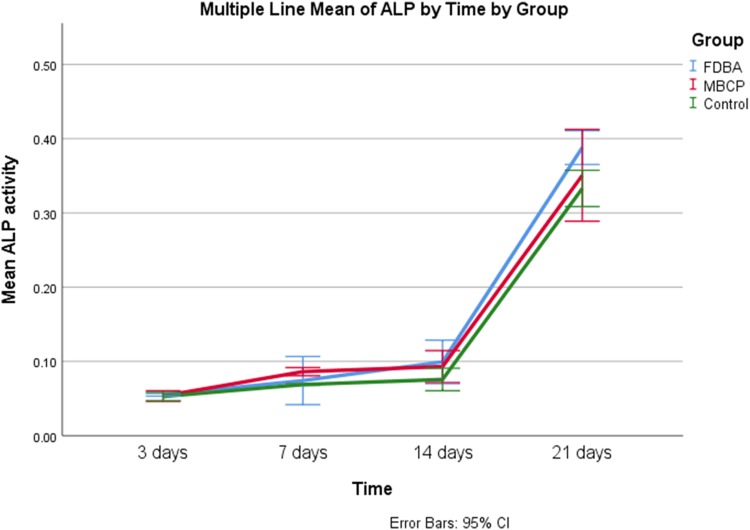
Comparison of mean ALP activity between study groups cultured in the standard medium at different time points

In the standard medium, the group (*p* = 0.001) and the time (*p* < 0.001), as well as their interaction (*p* = 0.028), had a significant effect on ALP activity. Figure 6 shows that ALP activity increased significantly between time points (*p* ≤ 0.001) except between day 7 and 14 of culture (*p* = 0.137). There was no significant difference in ALP activity of FDBA and MBCP group’s culture in the standard medium (*p* = 0.374), while ALP activity of both groups was higher than the control group (*p* < 0.05).

**Fig. 7:**
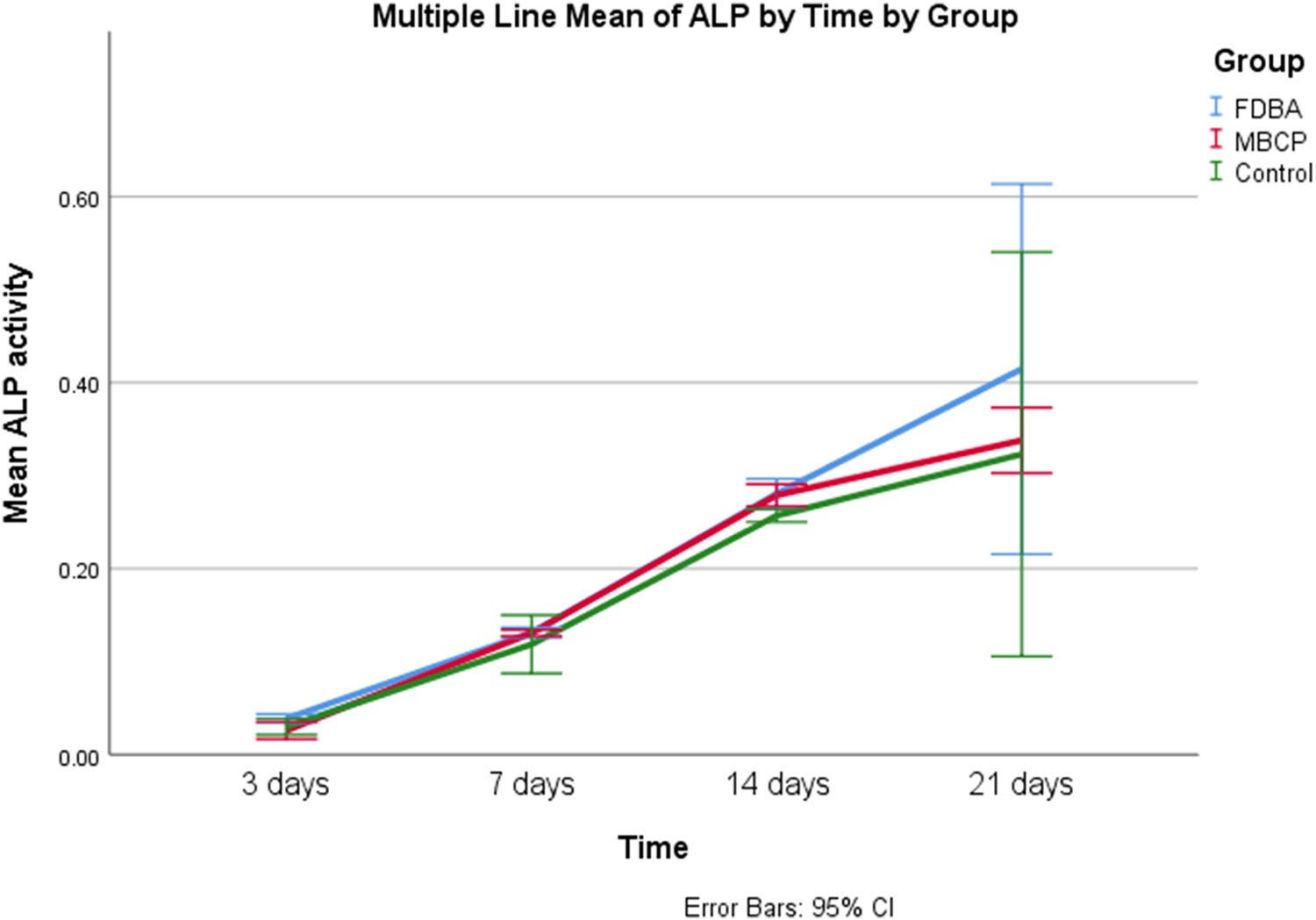
Comparison of mean ALP activity between study groups cultured in the osteogenic medium at different time points

In the osteogenic medium, the group (*p* = 0.001) and the time (*p* < 0.001), as well as their interaction (*p* = 0.042), had a significant effect on ALP activity. Figure 7 shows that ALP activity was increased significantly between experiment time points (*p* < 0.001). FDBA group had significantly higher ALP activity compared to MBCP (*p* = 0.035) and control (*p* = 0.001) groups. There was no significant difference between ALP activity of MBCP and control groups (*p* = 0.645).

## Discussion

The current in vitro study was performed to compare the response of DPSCs to two types of commonly used bone granules. The results revealed that although both materials could enhance attachment, proliferation, and differentiation of DPSCs, FDBA could better support stem cells compared to synthetic biphasic HA/β-TCP granules (MBCP).

MBCP is a synthetic biphasic bone substitute material including 80% β-TCP and 20% HA and it has been reported to have 60–70% porosity with 300–600 µm pore size (Arinzeh et al. 2005, Cordonnier et al. 2014). Our measurements showed that MDBP granules had 350–700 µm macropores which did not seem to be interconnected as well as 2–20 µm micropores while FDBA granules had relatively few 50–500 µm pores. Based on in vitro study by Kim et al. (2017), proper pore size for attachment of osteoblasts is 400–700 µm. Other factors such as surface design could influence attachment of cells as well. Bowers et al. (1992) demonstrated that osteoblasts attach more on rough surfaces than on smooth surfaces.

In the current study, SEM images showed an increase in the attachment of DPSCs to both granules from day 1 to day 7 of culture. For FDBA granules, the attached cells seemed to be wider and producing more ECM compared to MBCP granules (Fig. 4). However, cell attachment was more probable on rough edges of FDBA granules. A previous study also showed attachment of clusters of long cells penetrating into scaffold pores and attached to its rough edges and formation of collagen-like fibers on FDBA granules (Motamedian et al. 2017). Similarly, (Tang et al. 2002) showed penetration and attachment of mesenchymal stem cells (MSCs) to FDBA pores. Studies by (Cordonnier et al. 2010, 2014) on MBCP bone granules showed proper attachment of MSCs during the first day and production of ECM after 21 days.

The number of attached cells on both scaffolds increased during the 7-day experiment. This finding which shows the biocompatibility of these materials was in agreement with previous studies on similar granules (Cordonnier et al. 2010; Seebach et al. 2010; Lafzi et al. 2016; Motamedian et al. 2017). Similar to cell attachment, the proliferation of DPSCs was also higher on FDBA granules in the current study. Previous studies have also shown high proliferation of cells on FDBA granules compared to demineralized FDBA (Lafzi et al. 2016) and xenograft (Motamedian et al. 2017).

Alkaline phosphatase is the marker of choice for assessing the maturity of osteogenic cells (Golub et al. 1992). In the current study, ALP activity of DPSCs culture on both granules increased during the 21-day experiment. ALP activity is associated with osteopontin which is a late-stage osteoblast development marker (Beck et al. 1998). Therefore, the increase in ALP activity which occurred during the second week in the osteogenic medium and during the third week in the standard medium could indicate osteoblastic differentiation. A noteworthy finding was the ability of both materials to elevate ALP activity in the standard medium compared to the control group. This finding shows in vitro osteoinductive property of these granules. However, in the osteogenic medium, there was no difference in ALP activity of MBCP and control groups while it was higher in FDBA group. This could indicate that the osteoinductivity was more dominant in FDBA granules compared MBCP ones. Motamedian et al. (2017) had shown that osteogenic genes were expressed by DPSCs cultured with FDBA after 1 week in both standard and osteogenic media which was higher than xenograft and synthetic β-TCP. Similarly, previous studies on biphasic HA/TCP granules had shown an increase in ALP activity (Arinzeh et al. 2005; Cordonnier et al. 2010). Arinzeh et al. (2005) demonstrated that HA/TCP granules elevate ALP activity of hMSCs after 4 weeks, but not sooner than that.

The results of the current study could be interpreted considering the applications of these bone granules. In clinical situations, the clinician would use bone substitutes to fill the defect. A proper material would provide a matrix for local stem cells and progenitor cells to initiate osteogenesis. In addition, in classic bone tissue engineering, bone substitute materials and scaffolds are used to provide a bed for attachment of stem cells. The ex vivo engineered graft in this method would be transferred to the defect site. Therefore, considering the results of this study, it could be suggested that as allografts had a better attachment, proliferation, and differentiation of stem cells, they might be better bone substitute materials for both aforementioned applications compared to biphasic synthetic granules. However, it should be considered that this in vitro comparison might not be in agreement with in vivo situations. In vitro studies could eliminate the effect of confounding factors and help assessment of a desired factor. Also, evaluation of cell proliferation and differentiation is easier in these first-line studies. In vitro studies only can suggest proper material for bone regeneration, because several in vivo conditions such as recipient site, body fluids, and immune system were not considered. Therefore, further animal and clinical comparison of bone materials are inevitable.

## Conclusion

Considering the limitations of this in vitro study, it could be concluded that both materials were biocompatible and were able to induce alkaline phosphatase (ALP) activity in both standard and osteogenic cell culture media. Freeze-dried bone allograft (FDBA) granules had better attachment and higher proliferation and ALP activity of dental pulp stem cells compared to biphasic hydroxyapatite/beta-tricalcium phosphate bone granules. Therefore, FDBA granules could serve as a better vehicle for carrying stem cells or proper bone substitute material which could induce proliferation and differentiation of local stem cells. Further in vivo studies are required.
